# Effect of Herbal Intracanal Medicaments on Microhardness of Root Dentin: An In Vitro Study

**DOI:** 10.7759/cureus.63165

**Published:** 2024-06-25

**Authors:** Gaurav Patri, Harshita Lath, Debkant Jena, Aanchal Banka, Atul Anand Bajoria

**Affiliations:** 1 Department of Conservative Dentistry and Endodontics, Kalinga Institute of Dental Sciences, Kalinga Institute of Industrial Technology (Deemed to be University), Bhubaneswar, IND; 2 Department of Oral Medicine and Radiology, Kalinga Institute of Dental Sciences, Kalinga Institute of Industrial Technology (Deemed to be University), Bhubaneswar, IND

**Keywords:** intracanal medicament, herbal, dentin microhardness, curcumin, chitosan, aloe vera

## Abstract

Introduction: The use of intracanal medicaments (ICM) is crucial to reduce the microbial load in root canals. However, its application may negatively affect the physical properties of root dentine. Thus, this in vitro study was performed to investigate the effects of different herbal ICM on the microhardness of root dentin.

Materials and methods: A total of 100 mid-root discs were randomly divided into five groups (n = 20) and treated with Aloe vera, Aloe vera + chitosan, curcumin, curcumin + chitosan, and control for 14 days. A Vickers hardness indentation machine (Laizhou Laihua Testing Instrument Factory, Yantai, China) with a load of 200 g and a dwell time of 15 seconds was used for microhardness testing before and after treatment. The results were analyzed using Statistical Product and Service Solutions (SPSS, version 26.0; IBM SPSS Statistics for Windows, Armonk, NY). Intragroup comparisons were executed using paired t-tests, while intergroup comparisons employed ANOVA, followed by post-hoc Tukey's tests.

Results: The Aloe vera + chitosan and curcumin + chitosan groups showed a statistically significant reduction in dentin microhardness (p < 0.05). The decrease in dentin microhardness of the Aloe vera and curcumin groups was non-significant (p > 0.05) and similar to that of the control.

Conclusion: Aloe vera or curcumin alone as ICM did not affect the root dentin microhardness. The addition of 0.2% chitosan to either Aloe vera or curcumin negatively affected the root dentin microhardness.

## Introduction

The successful outcome of endodontic treatment hinges on the eradication of microbial pathogens and their byproducts from the root canal system [1]. Utilizing instruments and solutions during cleaning and shaping procedures is crucial in ensuring the elimination of a large quantity of bacteria and their toxins [1,2]. The complex nature of root canal systems renders it unfeasible to solely rely on cleaning and shaping techniques to reduce bacterial populations and establish a sterile environment [2-5]. To further diminish microbial populations, the application of root canal medicaments is a significant measure to undertake [2,6].

Intracanal medicaments (ICM) are extensively employed in multi-session endodontic treatments to reduce microbial proliferation between visits. Their main role is to sterilize the root canal system, especially when mechanical cleaning fails to eradicate persistent bacteria that survive between sessions. Additionally, these agents target remaining microorganisms and block the entry of pathogens resulting from defective restorations [7,8].

The most commonly employed medicament in modern endodontic procedures is calcium hydroxide (CH), as other options such as formacresol, phenols, paraformaldehyde, iodoform, and antibiotics (e.g., tetracycline, metronidazole), have been linked to biological toxicity, allergic responses, and resistance [2,3]. CH is recognized for its antibacterial, anti-inflammatory, and osteogenic properties [9]. Unlike conventional medicaments such as aldehydes and phenols, CH does not cause notable systemic or local side effects [10,11]. Owing to its elevated pH, CH is effective in eradicating certain bacteria from the root canals within seven days but is ineffective against Enterococcus fecalis, a frequently encountered bacteria in failed endodontic treatments [1]. Moreover, prolonged use of CH as an intracanal medicament has been reported to negatively impact the microhardness of root dentin [6,10,11].

The potential adverse effects associated with CH have sparked a growing interest in natural alternatives for endodontic medicaments. These alternatives encompass a polyherb (triphala) containing Emblica officinalis (amla), Terminalia belerica (baheda), and Terminalia chebula (chebulic myrobalan), chitosan, Aloe barbadensis Miller (Aloe vera), Curcuma longa (turmeric), Allium sativum (garlic), propolis (bee glue), Morinda citrifolia (Noni), Acacia nilotica (gum Arabic tree or Babul), Salvadora persica (miswak) solution, Camellia Sinensis (green tea) polyphenols, and more [12]. Herbal products are valued for their biocompatibility, potent anti-inflammatory, good antibacterial, and antioxidant properties [12,13].

Curcumin, a key component of Curcuma longa, contributes significantly to its biological properties [14-16]. Among its various therapeutic benefits, curcumin displays strong antimicrobial and antiviral effects, inhibiting bacterial cell division and proving efficacy against E. faecalis [14,16]. Aloe vera extract showcases antibacterial traits attributed to anthraquinones and numerous active constituents, making it a biocompatible and minimally toxic intracanal medicament [2,14].

One essential requirement for an intracanal medicament is its stability and sustained release over an extended period. Chitosan, a biopolymer derived from chitin deacetylation, serves as a suitable drug carrier that facilitates gradual and controlled medicament release [9]. It also exhibits bio-adhesive properties and low toxicity, enhancing the antibacterial efficacy of ICM [9]. While there are conflicting findings regarding the antibacterial effectiveness of Aloe vera against E. faecalis, recent research has demonstrated improved antimicrobial outcomes when Aloe vera and curcumin are combined with chitosan as a carrier [14,17].

The microhardness of root dentin is frequently linked to its mineral content, where lower microhardness suggests demineralization and higher microhardness indicates better mineralization [1]. A decrease in root dentin microhardness results in a softer dentin structure, adversely affecting the sealing efficiency of obturation materials [2]. This compromise in sealing can negatively impact the outcome of endodontic treatments and may increase the risk of root fractures [1,10]. Given the challenge of preserving tooth microhardness and achieving complete or near-complete eradication of microbes within root canals, there is a necessity to identify the most effective intracanal medicament capable of addressing both aspects [2].

The use of curcumin and Aloe vera alone as ICM does not have a discernible effect on root dentin microhardness [2,15]. Limited literature exists on the impact of combining Aloe vera and curcumin with chitosan on root dentin microhardness. Thus, this study seeks to assess and compare the influence of this amalgamation on root dentin microhardness at various time points. The null hypothesis posits that the administration of these test agents as ICM will not yield any significant alterations in root dentin microhardness.

## Materials and methods

This study was undertaken in the Department of Conservative Dentistry and Endodontics, Kalinga Institute of Dental Sciences, Bhubaneswar, after obtaining approval from the Institutional Ethical Committee (KIIT/KIMS/IEC/892/2022).

Sample size estimation and sampling technique

The sample size was calculated using G*Power software (version 3.1.9.7; The G*Power Team, Germany) with an alpha error probability of 0.05 and a power (1−β error probability) of 0.8. It was estimated that a minimum overall sample size of 90, with 18 subjects per group, would be adequate for an alpha level of 0.05 and a confidence interval of 95%. Thus, to round off the samples in each group, 20 samples per major group and 10 samples for a subgroup were selected. Thus, the total final sample size was 100, and a simple random sampling method was used to allocate teeth to respective groups.

Inclusion and exclusion criteria

In this investigation, root dentin slices were obtained from teeth extracted due to advanced periodontitis or for therapeutic reasons in orthodontics to alleviate crowding or to improve alignment. The samples comprised mature, permanent, single-rooted teeth (mandibular or maxillary), from individuals aged between 18 and 40 years. Teeth exhibiting resorption, decay, or fractures extending below the cementoenamel junction, as well as those with developmental anomalies or prior root canal treatments, were excluded from the study [1].

Preparation of dentin discs

Haapasalo et al.'s [18] proposed model underwent modifications for this study, which utilized 50 freshly extracted human single-rooted anterior or premolar teeth with intact cementum. Following the removal of debris, calculus, and soft tissue remnants using an ultrasonic scaler (DentGist, Woodpecker, China), the teeth were stored in 0.1% thymol at room temperature. Subsequently, the teeth were decoronated with a diamond disc (NMD Nexus Medodent, India), and the pulp was extirpated using a #10 k-file (Mani, Japan). The same file was passed passively till it was visible at the apex of the root, and the working length was established by substracting 0.5 mm from the total length and was reconfirmed radiographically using a RVG (Vatech Co., South Korea). Biomechanical preparation was done using the Protaper Universal system (Dentsply Maillefer, Switzerland) up to File F3, with irrigation by 3% sodium hypochlorite (Prime Dental, India). The roots were then immersed in acrylic resin and sectioned transversely into coronal, middle, and apical thirds with a hard tissue microtome (Leica Biosystems, Germany), which produced polished and equal sections. The apical and coronal sections were discarded, and a 4 mm segment from the middle third of each root was utilized to create two discs of root dentine having a thickness of 2 mm [1].

Preparation of medicaments

Curcumin Extract Preparation: 2 mg/mL

**Figure 1:**
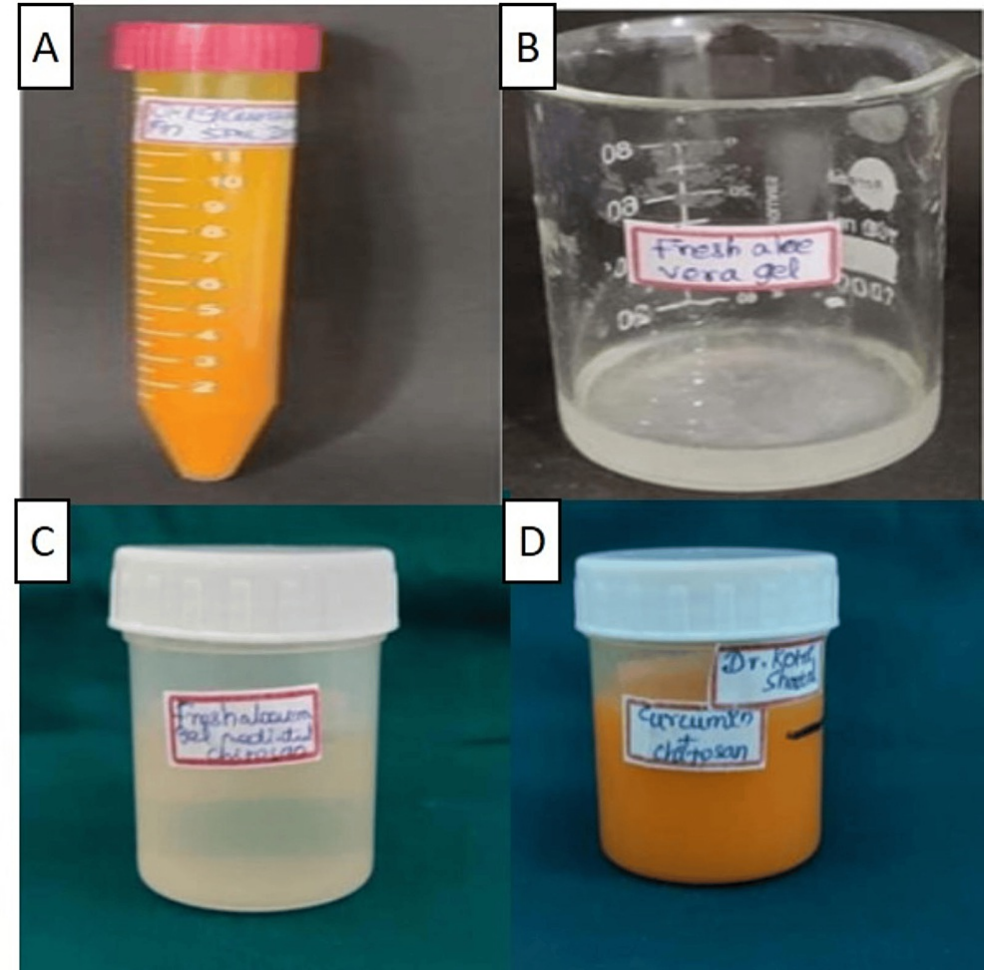
Test medicaments 1A: Curcumin; 1B: Aloe vera; 1C: Aloe vera/Chitosan; 1D: Curcumin/Chitosan

Specifically, 20 mg of curcumin (Himedia, India) was dissolved in 5 mL dimethyl sulphoxide (Sigma Aldrich, India), and then this mixture was added to 5 mL distilled water to make 10 mL of curcumin extract (Figure 1A). The prepared extract was kept in a magnetic stirrer (Sigma Aldrich, India), at 700 rpm for 48 hours [14].

Aloe vera Gel

Thick succulent leaves of Aloe vera (Aloe barbadensis) plant were obtained from Medicinal plant's garden, Dr. Abhin Chandra Homoeopathic Medical College and Hospital, Bhubaneswar. To obtain Aloe vera extract (Figure 1B), the mucilaginous gel from the inner parenchyma of the Aloe vera plant leaves was used. The leaves were rinsed with water and then cut into transverse sections. Using a vegetable peeler, the thick outer skin was carefully removed, and the central gel-like pulp was scooped out with a spoon. Additionally, 10 g of the scooped gel was uniformly crushed using mortar and pestle to form a gel-like consistency [19].

Aloe vera/Curcumin With 0.2% Chitosan Preparation

Specifically, 200 mg of chitosan((Himedia) was measured and dissolved with 100 mL of 1% glacial acetic acid (Sigma Aldrich, India). The resultant solution was kept in a magnetic stirrer at 700 rpm for 48 hours [14]. All test agents that were in solution form were magnetically stirred to thicken them to a gel-like consistency for uniformity (Figures 1C, 1D).

Experimental procedure

**Figure 2:**
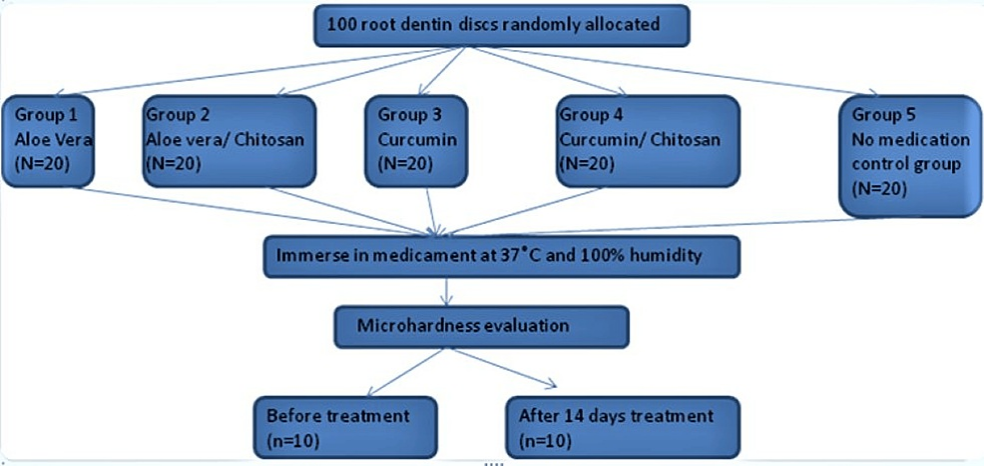
Diagrammatic illustration of the groups and experimental procedure

A total of 100 root dentin discs, after preparation, were subsequently randomly allocated into five distinct groups (Figure 2) as follows: Group 1 received Aloe vera treatment, Group 2 was subjected to Aloe vera/chitosan intervention, Group 3 underwent curcumin application, Group 4 received curcumin/chitosan treatment, and finally Group 5 served as the control group with no medication administered. The specimens were immersed in the ICM, as per the designated groups, enclosed in leakproof containers, and maintained at a stable temperature of 37°C with 100% humidity in an incubator. Subsequently, these specimens were subjected to microhardness evaluation both before and 14 days after the medicament application, with 10 samples being analyzed at each specific time point [1,2].

Microhardness evaluation

**Figure 3:**
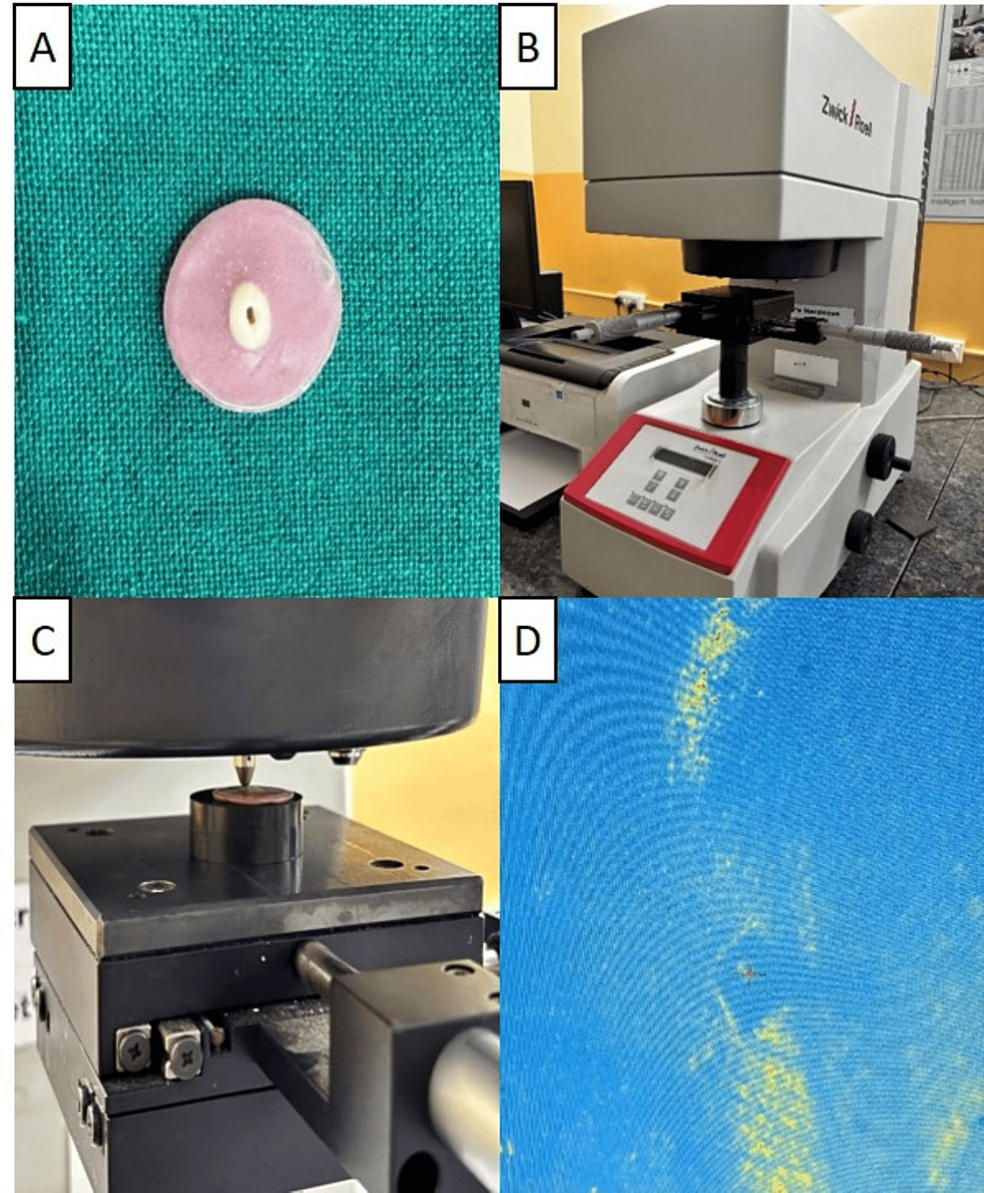
Microhardness testing 3A: Dentin sample for microhardness testing; 3B: Vickers hardness tester (ASTM E 384; Zwick Roell, India); 3C: Microhardness testing of the dentin sample; 3D: Microscopic view of indentation

Following the administration of the respective medicaments, the specimens underwent a thorough rinsing procedure using distilled water and were subsequently dried employing absorbent paper before the microhardness assessment. The microhardness evaluation was conducted both before and 14 days after the application of the medicaments utilizing a Vickers hardness indentation machine (ASTM E 384; ZwickRoell, India). At a magnification of 10X, the indentations were executed, with three distinct indentations being recorded at a depth of 100 µm each, positioned 1 mm away from the walls of the root canal (Figure 3). This process involved the application of a 200 g load and a 15-second dwell time, following which the mean value was meticulously calculated. The results were documented as the Vickers hardness number (VHN). The entire microhardness testing procedure, encompassing measurements and data recording on the data collection form, was executed by an examiner who was blinded to the experimental conditions [1].

Statistical analysis

Data entry was completed using MS Excel (MS Office version 2009; Microsoft, Redmond, WA). Descriptive statistical analyses were conducted to determine the mean and standard deviation. Intragroup comparisons were executed using paired t-tests, while intergroup comparisons employed ANOVA, followed by post-hoc Tukey's tests. Statistical significance was defined at a p-value threshold of <0.05. All statistical analyses were performed using Statistical Product and Service Solutions (SPSS, version 26.0; IBM SPSS Statistics for Windows, Armonk, NY).

## Results

**Figure 4:**
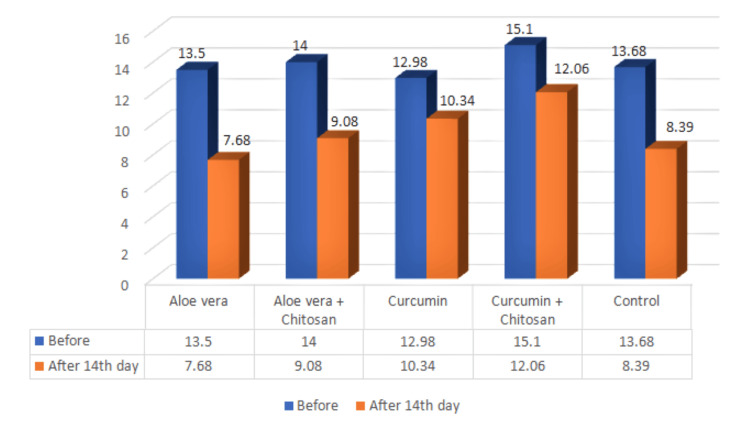
Mean effect of intracanal medicaments on microhardness of the root dentin

Table 1 and Figure 4 provide the descriptive data of the root dentin microhardness (VHN) in both pretreatment and posttreatment. Table 1 also provides the data on intragroup comparison using paired t-tests. Based on the data obtained at baseline and after 14 days, it was observed that a statistically significant difference (p < 0.05) was seen for group II (14.00±2.44/9.08±2.36) and group IV (15.10±9.58/12.06±8.47), pre- and posttreatment, while a non-significant difference (p > 0.05) was observed for group I (13.50±2.32/7.68±1.98), group III (12.98±10.89/10.34±7.43), and group V (13.68±10.88/8.39±9.56) pre- and posttreatment.

**Table 1:** Mean effect of intracanal medicaments on microhardness of the root dentin with intragroup comparison using the paired t-test * p value <0.05 was considered statistically significant.

Groups evaluated	Evaluation period	Mean (VHN)	Standard deviation (VHN)	P value (sig.)
Group I (Aloe vera)	Before	13.50	2.32	>0.05
	After 14 days	7.68	1.98	
Group II (Aloe vera + chitosan)	Before	14.00	2.44	<0.05*
	After 14 days	9.08	2.36	
Group III (Curcumin)	Before	12.98	10.89	>0.05
	After 14 days	10.34	7.43	
Group IV (Curcumin + chitosan)	Before	15.10	9.58	<0.05*
	After 14 days	12.06	8.47	
Group V (Control)	Before	13.68	10.88	>0.05
	After 14 days	8.39	9.56	

Table 2 denotes the intergroup comparison amongst the test groups employing ANOVA and pairwise comparison using Tukey’s post-hoc test. A statistically significant difference (p < 0.005) was observed when group I was compared to groups II and IV; group II was compared to groups I, III, and V; group III was compared to groups II and IV; and group IV was compared to groups I, III, and V. Group V (control) showed a statistically significant difference (p < 0.005) when compared with groups II and IV. Comparison between groups I with group III and group V showed non-significant results (p > 0.05). Similarly, group II and group IV comparisons also showed non-significant results (p > 0.05).

**Table 2:** Intergroup comparison of the effect of intracanal medicaments on microhardness of the root dentin using the ANOVA and Tukey’s post-hoc tests * p value <0.005 was considered statistically significant.

(I) Group	(J) Group	Mean difference (I-J)	p-value	95% ConfidenceiInterval	
				Lower Bound	Upper Bound
Group I (Aloe vera)	Group II	-1.40	<0.005*	-0.1907	0.0657
	Group III	-2.66	1	0.1588	0.4152
	Group IV	-4.38	<0.005*	0.1580	0.4145
	Group V	-0.71	0.076	-0.2213	-0.4770
Group II (Aloe vera + chitosan)	Group I	1.40	<0.005*	-0.0657	0.1907
	Group III	-1.26	<0.005*	0.2213	0.4777
	Group IV	-2.98	0.087	0.2205	0.4770
	Group V	0.69	<0.005*	-0.1580	-0.4770
Group III (Curcumin)	Group I	2.66	0.084	-0.4152	-0.1588
	Group II	1.26	<0.005*	-0.4777	-0.2213
	Group IV	-1.72	<0.005*	-0.1290	0.1275
	Group V	1.95	0.056	-0.4770	-0.2205
Group IV (Curcumin + chitosan)	Group I	4.38	<0.005*	-0.4145	-0.1580
	Group II	2.98	0.34	-0.4770	-0.2205
	Group III	1.72	<0.005*	-0.1275	0.1290
	Group V	3.67	<0.005*	-0.4152	0.1290
Group Group V (Control)	Group I	0.71	0.075	-0.4145	-0.1580
	Group II	-0.69	<0.005*	-0.4770	-0.2205
	Group III	-1.95	0.067	-0.1275	0.1290
	Group IV	-3.67	<0.005 *	-0.4152	0.1290

## Discussion

In this in vitro investigation, the impact of herbal remedies (specifically Aloe vera and curcumin) both separately and in conjunction with chitosan as a carrier on the microhardness of root dentin was assessed before treatment and after 14 days. Existing literature has highlighted the limitations of relying exclusively on chemo-mechanical methods for cleaning the root canal, resulting in diminished antimicrobial efficacy [6]. Consequently, the inclusion of ICM in endodontic therapy has become imperative, serving as an intermediary dressing between appointments [8,20]. Various studies have documented instances of demineralization, surface deterioration, and weakened mechanical characteristics of radicular dentin due to prolonged exposure to ICM, ultimately leading to the formation of microcracks and vertical root fractures [15,21]. In response to these challenges within endodontics, recent attention has been directed towards the utilization of biologically derived medications sourced from natural plants, with Aloe vera and curcumin standing out for their notable antibacterial properties [14,15]. Given the ongoing pursuit for an optimal ICM that can effectively disinfect the root canal system while preserving the structural integrity of the root dentin, coupled with the paucity of literature examining the individual effects of Aloe vera and curcumin on root dentin microhardness and the absence of data on the combined impact of chitosan with Aloe vera or curcumin, the rationale for undertaking this study becomes apparent.

Microhardness assessment has been validated as a means to indirectly support the changes in mineral content in mineralized dental tissues, correlating with the quantity of calcified matrix per square millimeter [1,21]. The utilization of microhardness testing has been identified as the most feasible approach for indirect quantitative evaluation, enabling the measurement of demineralization in dental hard tissues [21]. The examination of microhardness offers valuable insights into the interactions of dentin with various therapeutic agents [21]. In this particular investigation, Vickers hardness testing was utilized due to its sensitivity to measurement inaccuracies, resilience to surface variations, and capability to accurately assess small specimens such as root discs [20,22].

The tubular density of dentin has been observed to elevate as one progresses from the cervical to the apical regions of radicular dentin, leading to a negative correlation between radicular dentinal microhardness and radicular tubular density. Such variations in the adjacent regions of dentinal tissue may potentially induce modifications in the outcomes. Therefore, the current investigation conducted a microhardness assessment specifically within the middle-third section of the root architecture in each specimen [1,21]. Based on the results obtained the null hypothesis was rejected as the microhardness of root dentin in all the test groups decreased significantly or insignificantly.

Upon examination of the data in intragroup comparison (Table 1), it was determined that groups I, III, and V displayed a non-significant difference (p > 0.05) between pre and post-treatment. These results align with previous research conducted by Parashar V et al [2], Prabhakar AR et al [15], and Sinha et al [23]. Parashar V et al [2], noted in their study that the Aloe vera group exhibited the least reduction in microhardness compared to other treatment groups. Sinha et al [23], in a randomized control trial, reported that Aloe vera did not have any detrimental effects on the shear bond strength of dentin when utilized as a cavity disinfectant. Prabhakar AR et al, in their investigation, concluded that Turmeric extract demonstrated significant antibacterial properties without impacting the microhardness of root dentine [15]. It can be argued that Aloe vera and curcumin, as natural plant extracts free from synthetic agents, do not produce adverse effects on root dentin.

Upon analysis of the outcomes, a notable decrease in root dentin microhardness (p < 0.05) was observed within groups II and IV before and after the respective treatments, as revealed by intragroup comparison (Table 1). The current findings stand as pioneering in nature, as existing literature lacks supportive evidence on this matter. The detrimental impact on root dentin resulting from the introduction of 0.2% chitosan to Aloe vera or curcumin was evident. Nikhil et al. [24] reported that using 0.2% chitosan, 1% phytic acid, and 17% ethylenediaminetetraacetic acid (EDTA) resulted in reduced dentin microhardness. In a similar vein, Pimenta et al. [25] found that EDTA, citric acid, and chitosan all decreased root canal microhardness, with no statistically significant differences observed between these solutions. The presence of chitosan as a natural chelator could explain this outcome [24]. Chelation can modify the calcium-to-phosphorus ratio in dentin, reducing its mineral and non-collagenous protein (NCP) content and, thus, likely lowering microhardness while increasing the permeability and solubility of root canal dentin [24]. Despite the widespread application of chitosan, its exact mechanism of action remains largely unknown. The hypothesis suggests that the hydrophilic nature of chitosan polymer facilitates close interaction with root canal dentin, leading to its adsorption onto the canal walls. Furthermore, its cationic properties support ionic interactions between the calcium ions in dentin and the chelating agent. Given the insolubility of chitosan in water and the use of glacial acetic acid for solution preparation, the acidic environment may have augmented the chelating effectiveness of chitosan [24]. Additionally, considering that chelators are typically recommended for shorter durations, the time frame employed in the study likely contributed to the noteworthy decrease in root dentin microhardness [24].

Upon analyzing the outcomes through intergroup comparison (Table 2), it was observed that there was no statistically significant difference (p > 0.05) between Aloe vera and curcumin when assessed individually. These results closely resembled those of the control group (no medicament). Previous studies by Parashar et al. [2], Sinha et al. [23], and Prabhakar et al. [15] have supported and reinforced these findings. The rationale behind this similarity can be attributed to the inherent nature of Aloe vera and curcumin as pure plant extracts without any synthetic additives, thus preventing any adverse effects on the root dentin.

The findings of this research suggest that, while Aloe vera and curcumin did not harm the root dentin when used as an ICM, the inclusion of 0.2% chitosan had an adverse effect on the root dentin. Several limitations exist within this study. Firstly, it was conducted in vitro, utilizing extracted teeth, which may yield different outcomes compared to interactions within the oral cavity, especially in vital teeth displaying varying functions. Furthermore, results could have differed if the medicament had been applied throughout the entire root to the apex. The depth of penetration for each medicament was not assessed, nor were different time intervals considered. Future studies, both in vitro and in vivo, should address these limitations. Moreover, other characteristics of these medicaments such as biocompatibility, staining, and substantivity warrant further investigation.

## Conclusions

Within the scope of this investigation, it can be deduced that the root dentin microhardness was not influenced by either Aloe vera or curcumin. The introduction of 0.2% chitosan to Aloe vera or curcumin had a detrimental impact on the root dentin microhardness. Therefore, while the potential application of Aloe vera or curcumin as intracanal medicaments warrants further exploration, the addition of 0.2% chitosan is not recommended.
